# Indigenous people’s experiences of primary health care in Canada: a qualitative systematic review

**DOI:** 10.24095/hpcdp.44.4.01

**Published:** 2024-04

**Authors:** Geneveave Barbo, Sharmin Alam

**Affiliations:** 1 College of Nursing, University of Saskatchewan, Saskatoon, Saskatchewan, Canada; 2 Independent researcher, Montral, Quebec, Canada

**Keywords:** Indigenous people, primary health care, health services accessibility, systematic review, Canada

## Abstract

**Introduction:**

Indigenous people in Canada encounter negative treatment when accessing primary health care (PHC). Despite several qualitative accounts of these experiences, there still has not been a qualitative review conducted on this topic. In this qualitative systematic review, we aimed to explore Indigenous people’s experiences in Canada with PHC services, determine urban versus rural or remote differences and identify recommendations for quality improvement.

**Methods:**

This review was guided by the Joanna Briggs Institute’s methodology for systematic reviews of qualitative evidence. MEDLINE, CINAHL, PubMed, PsycInfo, Embase and Web of Science as well as grey literature and ancestry sources were used to identify relevant articles. Ancestry sources were obtained through reviewing the reference lists of all included articles and determining the ones that potentially met the eligibility criteria. Two independent reviewers conducted the initial and full text screening, data extraction and quality assessment. Once all data were gathered, they were synthesized following the meta-aggregation approach (PROSPERO CRD42020192353).

**Results:**

The search yielded a total of 2503 articles from the academic databases and 12articles from the grey literature and ancestry sources. Overall, 22 articles were included in this review. Three major synthesized findings were revealed—satisfactory experiences, discriminatory attitudes and systemic challenges faced by Indigenous patients—along with one synthesized finding on their specific recommendations.

**Conclusion:**

Indigenous people value safe, accessible and respectful care. The discrimination and racism they face negatively affect their overall health and well-being. Hence, it is crucial that changes in health care practice, structures and policy development as well as systemic transformation be implemented immediately.

**Highlights**

- This is the first qualitative systematic review to explore the experiences of Indigenous people with primary health care services across Canada.
- Following Joanna Briggs Institute’s systematic reviews of qualitative evidence methodology, this review included six academic databases as well as grey literature and ancestry sources.
- The experiences of Indigenous people accessing primary health care in Canada have been described as supportive and respectful in some cases, but also heavily included discriminatory attitudes and systemic challenges.
- Indigenous people living in rural or remote communities reported greater concern about privacy, confidentiality and accessibility compared to those residing in urban locations.

## Introduction

The 1946 Constitution of the World Health Organization (WHO) established that every human being has the fundamental right to the highest attainable standard of health.^1^ Nevertheless, to this day, health inequities continue to exist worldwide.^2^ Health inequities are systematic differences in the health status of various population groups caused by unequal distribution of social determinants of health that further disadvantage those who are already socially vulnerable.^2^,^3^ The WHO and other public health advocates assert the importance of investing in primary health care (PHC) as a means of addressing health inequities within countries.^4^,^5^

In Canada, PHC services have been offered to all eligible residents through the universal public health coverage, also known as Medicare.^6^ Medicare is governed by the 1984 *Canada Health Act*, which ensures the delivery of health care services (including PHC) and adherence to the five core principles of public administration, comprehensiveness, universality, portability and accessibility.^7^ In 2000, a PHC reform was agreed upon and launched by the federal, provincial and territorial governments, with the primary goal of improving service access, service quality and health equity as well as responsiveness to patients’ and communities’ needs.^6^,^8^ Yet, PHC access and quality issues continue to persist, particularly for socially marginalized populations, such as in the case of Indigenous Peoples.^9^,^10^ Social marginalization is often defined as social exclusion due to a lack of power, resources and status that leads to limited opportunity or accessibility.^11^

Numerous studies have highlighted barriers faced by Indigenous people who reside in urban and rural or remote locations when accessing PHC services, such as discrimination, racism, lack of culturally safe care and inaccessible care.^12^-^15^ Despite several qualitative accounts of these negative experiences, a deep search of the literature indicates that there still has not been a qualitative review conducted on this topic. Addressing this literature gap may assist policy makers, health care managers and professionals, and researchers in identifying key areas for improving PHC access and quality across Canada. 

Accordingly, we aimed to explore the following research questions:

- 1. What are the experiences and perspectives of Indigenous people with PHC services in Canada?
- 2. How do these experiences and perspectives differ when comparing PHC services provided in urban versus rural or remote settings?
- 3. What are the recommendations of Indigenous people to improve the quality of PHC services delivered in Canada?

## Methods

***Protocol and registration***

This systematic review is registered in the International Prospective Register of Systematic Reviews (PROSPERO CRD42020192353). 

***Eligibility criteria and search strategy***

Our review was guided by Joanna Briggs Institute (JBI) methodology for systematic reviews of qualitative evidence;^16^ the detailed protocol has been described elsewhere.^17^ English and French qualitative and mixed-methods articles were considered for inclusion if they focussed on first- or second-hand experiences of Indigenous people in Canada when receiving PHC services. There were no restrictions with respect to publication year or research participants’ age, gender, medical condition or geographical location.

A preliminary search of CINAHL and PubMed was conducted to identify keywords and terms relevant to the research questions. A complete search strategy was then developed and tailored to each selected database: MEDLINE, CINAHL, PubMed, PsycInfo, Embase and Web of Science (Table 1). Grey literature was also searched on Google Scholar, Bielefeld Academic Search Engine, ProQuest Dissertations and Theses and other relevant websites (e.g. Native Health Database and National Collaborating Centre for Indigenous Health). Furthermore, the reference list of each included article was examined to identify any additional studies for the review in order to obtain ancestry sources.

**Table 1:** Database search strategy

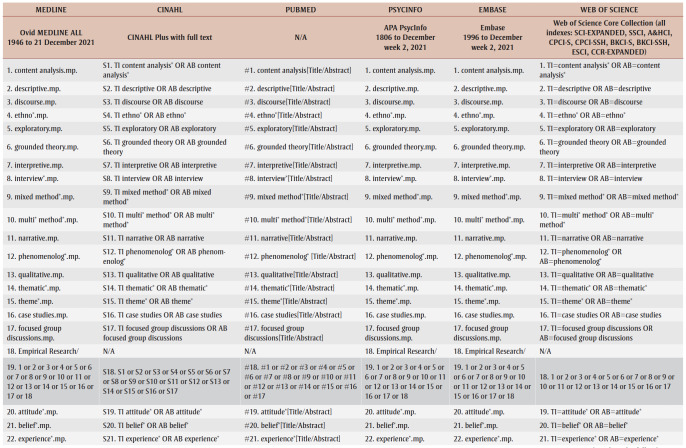 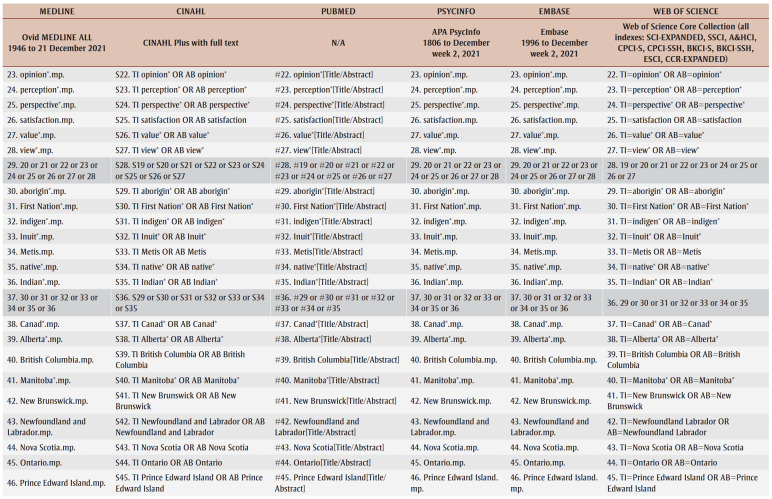 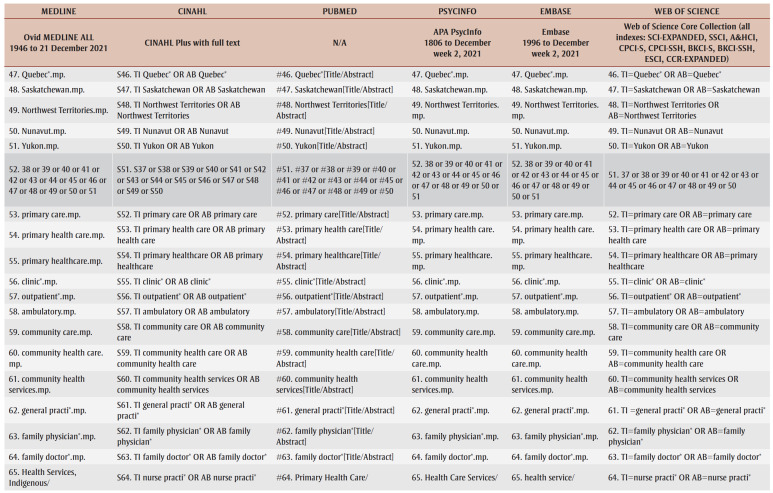 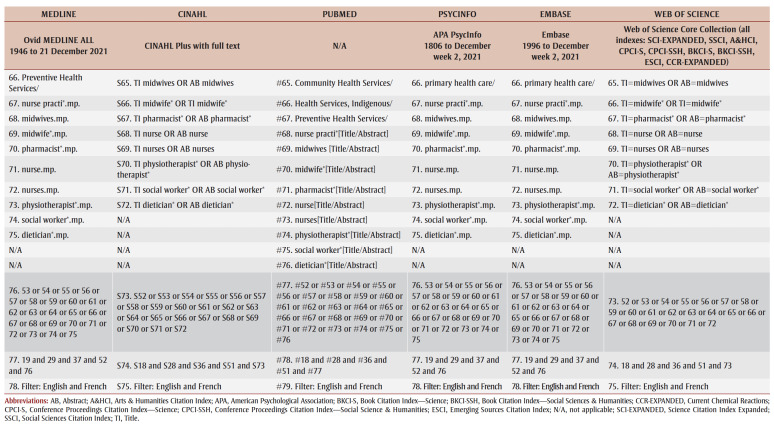

***Study selection***

Following the search, all identified citations were uploaded on Rayyan.^18^ Next, two authors (GB and SA) independently screened the articles’ titles and abstracts against the inclusion criteria. They then independently examined selected articles in full. Reasons for excluding certain articles were noted, and no major discrepancy arose between the two reviewers; hence, the assistance of a third reviewer was not needed. Once all included articles were identified, they performed an independent quality assessment using JBI’s Critical Appraisal Checklist for Qualitative Research.^19^

***Data extraction and synthesis***

All pertinent data from the included studies were then retrieved using the JBI data extraction tool.^16^ The extracted data included information on the studies’ methodology, approach to analysis, phenomena of interest, geographical location, participant characteristics, findings and illustrations. These data were then synthesized following JBI’s meta-aggregation approach; the findings and illustrations were aggregated into categories and further grouped together to create a comprehensive set of synthesized findings. Finally, consistent with Munn et al.,^20^ these synthesized findings were assigned a ConQual score to demonstrate their dependability and credibility.

## Results

The search yielded a total of 2503 articles from the academic databases and 12 articles from the grey literature and ancestry searches. Overall, 22 articles were included in this review. 

**Figure 1:**
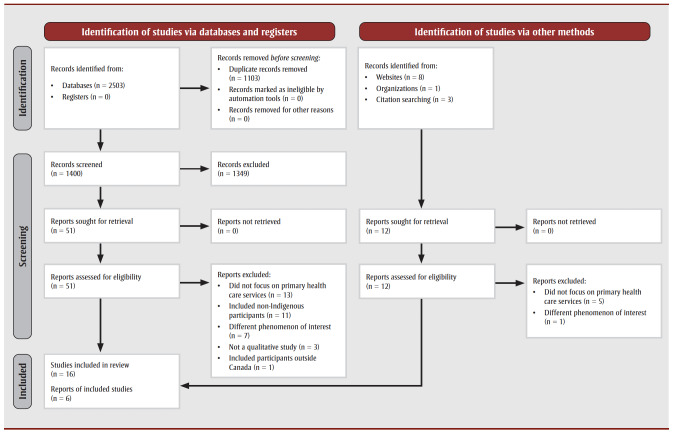
PRISMA 2020 flow diagram for new systematic reviews

Figure 1 illustrates the PRISMA flow diagram of the search results and study selection process.^21^ The methodological quality of all included articles was moderate to high; therefore, no studies were excluded following their appraisal (Table 2). 

**Table 2:** Assessment of methodological quality of included studies

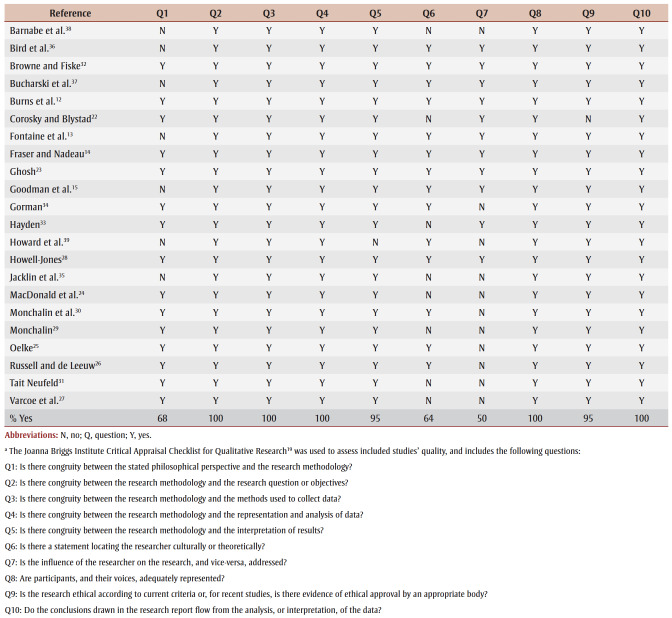

***Characteristics of included studies***

The detailed characteristics of the included studies are presented in Table 3. Articles were published between 2001 and 2020. Various qualitative approaches were used in these studies. These approaches included participatory research design,^12^,^22^-^27^ Indigenous methodologies,^13^,^15^,^24^,^28^-^31^ ethnography,^25^,^27^,^32^,^33^ phenomenology,^34^,^35^ case study,^14^,^36^ qualitative description,^12^,^37^ grounded theory^38^ and mixed methods.^39^ Eleven out of 22 studies represented experiences from major Canadian metropolitan areas, including Calgary,^25^,^38^ Edmonton,^37^ Ottawa,^23^ Toronto,^29^,^30^,^34^ Vancouver^15^,^28^ and Winnipeg,^13^,^31^ while 10studies were conducted in rural or remote communities within the provinces of British Columbia,^26^,^27^,^39^ Manitoba,^33^ Nova Scotia,^12^,^24^ Ontario^24^ and Quebec^14^ and within the Canadian territories of Nunavut^22^ and Northwest Territories.^32^ Finally, one article included findings from multiple provinces and locations, with participants from urban southern and rural Alberta, urban northern and remote northern Ontario, and rural British Columbia.^35^ The categorization of urban versus rural or remote settings was based on the study setting as defined by the authors as well as by the population density; urban areas are characterized as having at least 400 people per square kilometre, and the opposite is true (< 400/km^2^) for rural or remote regions.^40^

**Table 3:** Characteristics of included studies

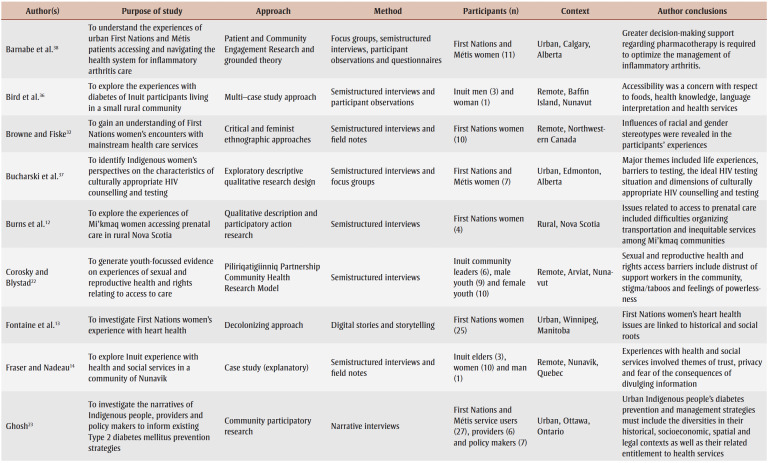 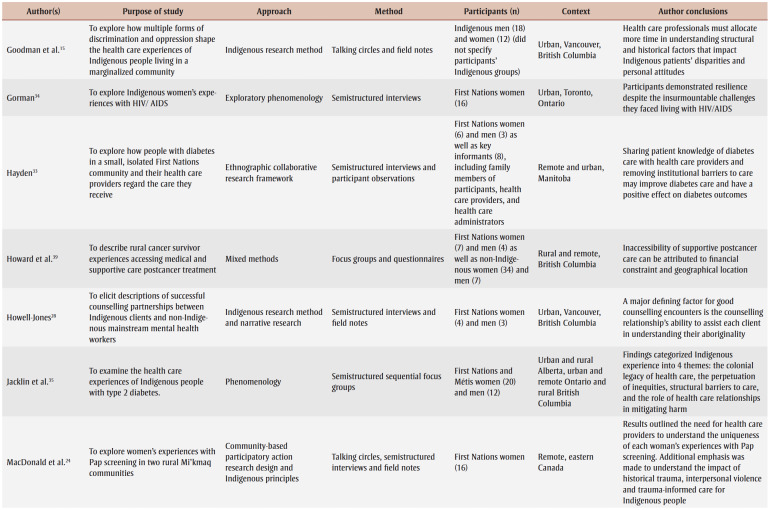 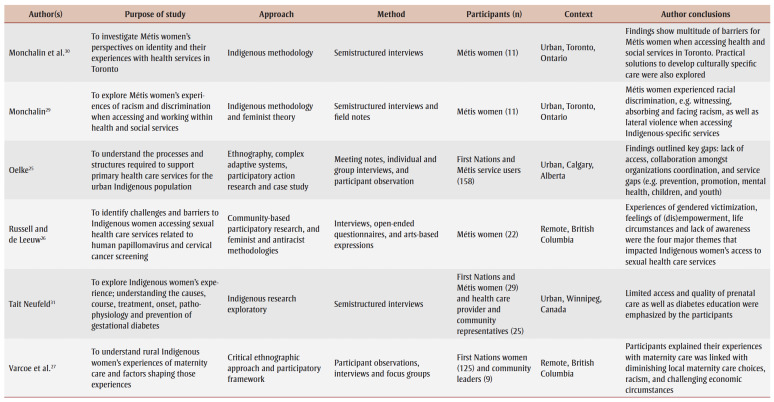

Research participants of included studies were from First Nations, Mtis and Inuit background, and overall were between the ages of 16 and 79 years. Their reasons for seeking PHC and their pre-existing medical conditions also varied (e.g. cancer, arthritis, diabetes, cardiovascular disease, human immunodeficiency virus and mental health disorders).

***Synthesized findings***

Table 4 presents an overview of the individual findings of our review. Three major synthesized findings emerged from these, pertaining to our first and second research questions, and another one arose for the third research question. Table 5 is a summary of findings containing each synthesized finding’s level of dependability and credibility, as well as ConQual score (which rates confidence in the quality of evidence from reviews of qualitative research) to help their evaluation and integration into education, practice and policy. 

**Table 4:** Results of metasynthesis of qualitative research findings

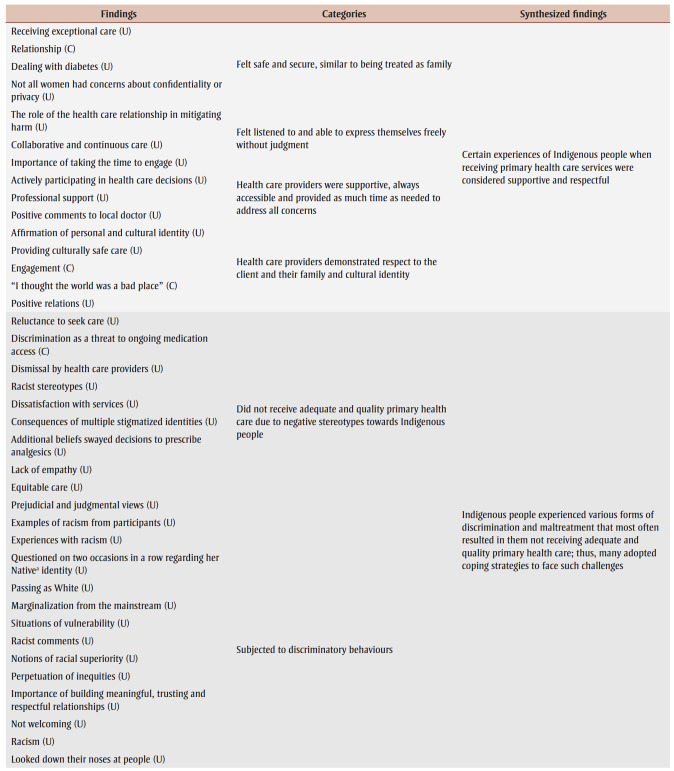 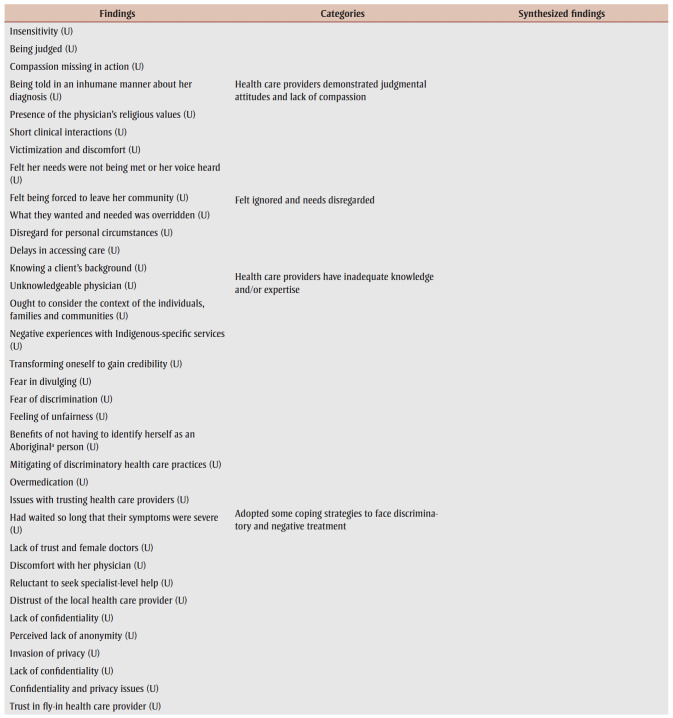 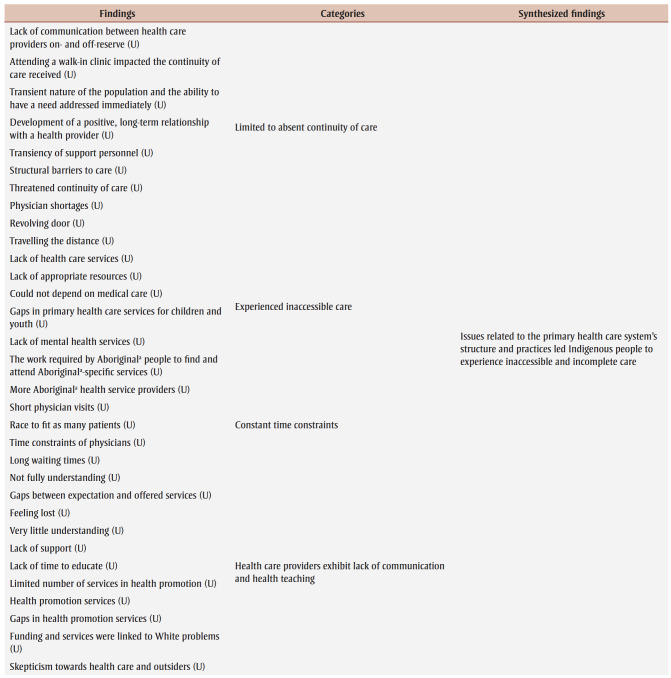 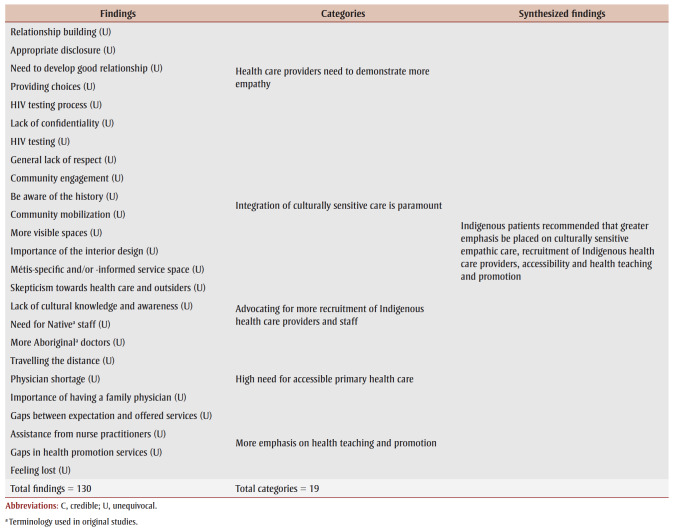

**Table 5:** Summary of findings

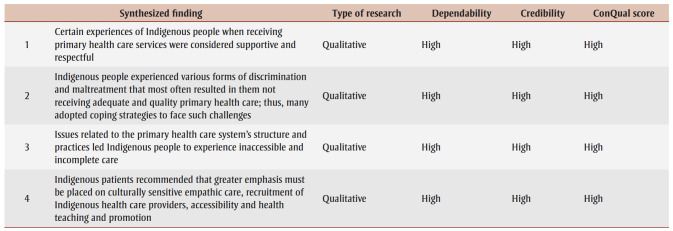

***Synthesized finding one: supportive and respectful experiences***

Synthesized finding one demonstrates that certain experiences of Indigenous people when receiving PHC were considered supportive and respectful. This metasynthesis was developed from four categories that included 15 findings. Some First Nations, Mtis and Inuit participants expressed that they had supportive and respectful encounters with PHC providers, as they felt safe, secure, listened to and freely able to express themselves without judgment. This finding was affirmed by one of the First Nations and Mtis participants living in an urban location as she described her prenatal care: “My G.P. is just a fantastic doctor because he sits there and actually listens to his patients. He respects that they know as much about what’s going on with their body as he probably does, if not more.”^31^^,p.165^ Another First Nations woman residing in a remote community echoed this positive experience: 

When my husband died, my [family] doctor phoned me to tell me to come in to talk with him and see if I was okay and talk about things that happened … and he explained it to me really softly; things like this happen. He was really caring. And that was the best thing that ever happened to me was him phoning me on his own to tell me that.^32^^,p.140^

Participants also greatly appreciated when PHC providers were supportive, accessible and offered as much time as needed to address all of their concerns; similar experiences were described by those residing in urban, rural and remote areas. Having access to dependable information and providers made a significant difference for many of the participants. Many First Nations women in rural communities said that their community health nurses were “always there” to assist them with their health needs.^12^

Moreover, Indigenous participants from urban, rural and remote locations valued health care providers demonstrating respect towards them, their family and their cultural identity. Providers were expected to exhibit culturally sensitive care and to have had training and to possess knowledge about Indigenous history, traditions, customs and challenges. When these qualities were present, PHC providers were perceived to be more helpful and genuine. Overall, across all settings—urban, remote and rural—instances of supportive and respectful PHC were experienced by First Nations, Mtis and Inuit participants.

***Synthesized finding two: discriminatory attitudes and maltreatment***

Synthesized finding two reveals that Indigenous people experienced various forms of discrimination and maltreatment that most often resulted in them not receiving adequate and quality primary health care; thus, many adopted strategies to cope with such challenges. Six categories and 58 findings were represented in this metasynthesis. 

There were numerous accounts in which participants shared their experiences of health care providers making comments or exhibiting behaviours based on discrimination. First Nations, Mtis and Inuit patients in urban and rural or remote areas were immediately assumed to have tobacco and drug addiction, to be intoxicated by alcohol, to have abusive partners, to mistreat their children, or any combination of these, without any actual justification or evidence of such claims.^13^-^15^,^25^,^34^,^38^ As reported by an aggravated Inuit participant from a remote community, “I arrived at the clinic and the first thing the doctor asked me is if I’m a smoker. Is that normal? It’s as if she assumed that because I’m Inuit I’m a smoker. I don’t think that is fair.”^14^^,p.293^ A First Nations woman in an urban setting also commented, “Oh I wouldn’t get the proper care if I needed it, like if I was in pain. They thought I’d be there just to get high.”^34^^,p.122^

These negative stereotypes automatically formed the basis of the care that Indigenous people received even though they did not necessarily apply to the specific situation of each patient. Consequently, these patients were generally dismissed, turned away and unable to receive the proper medical care they required, leading to severe complications or even death.^32^

Such situations were experienced in urban, rural and remote locations. As reported by a participant in Goodman et al.: 

I reached out on my right side and it really hurt. I went to a DTES [Downtown Eastside] clinic to the doctor and she told me to walk it off. I went to sleep and woke up and thought I was dying—big pain in my chest. I collapsed a lung. I think she thought I wanted painkillers, but I was really hurt.^15^^,p.90^

Another First Nations participant reported in Fontaine et al.:

I lost [a family member]. He did drink a lot. And anyway, he got sick and every time he went to the Nursing Station, the nurse in charge there told him, he said, “Oh, you have a severe hangover,” without checking him. And he went about three, I know three times for sure, whether the fourth time, I can’t remember. But anyway, they kept chasing him home, “There’s nothing wrong with you. You’re just ... quit drinking, get, you’re ... hung over,” you know. Anyway, he died one night in ... his home.^13^^,p.5^

Besides the deliberate omission of quality care, some Indigenous patients also sensed that certain PHC providers had discriminatory attitudes towards Indigenous people. In some cases, as soon as First Nations, Mtis and Inuit participants from both urban and rural or remote locations entered a clinic, they instantly felt unwelcomed and judged, based on how the health providers and staff looked at and talked to them. This was further extended in their subsequent interactions, as explained by one frustrated participant in Goodman et al.: 

So [the nurse] showed me how to [inject], but she was so mean about it. She was not accommodating. She said I should know how to do it myself. They treated me like crap, and I know it was because I was Native. We all know because of the look—there’s a look. When you need the medical care, we put up with it. We shouldn’t have to. We bleed the same way, we birth the same way. We have no choice …^15^^,p.89^

Some participants in urban as well as rural or remote areas thought that the negative attitudes and judgments of PHC providers may have stemmed from their lack of understanding or disregard for Indigenous life experiences, history, background and socioeconomic and political circumstances,^23^,^25^,^37^ but this was particularly emphasized by individuals living in rural or remote communities. There were instances in which First Nations women living on-reserve, who were required to travel to the city due to the unavailability of specialized services or diagnostic tools in their communities, were constantly fined for being late or missing their appointments in the city, even though the primary reasons for missing the appointments were that they were not able to afford a phone, or that there were traffic delays resulting from travelling a long distance.^32^ As Browne and Fiske reported, “The embarrassment associated with being late or with being asked to pay the cancellation fine when they lacked the money shaped women’s experiences and left women with the sense that they were being blamed for circumstances beyond their control.” 32^,p.138^

As a result of these various negative interactions with PHC providers and the health care system, numerous Indigenous patients learned to cope by deciding not to disclose their cultural identity and medical history, presenting themselves to look more credible, or simply avoiding seeking care. Certain participants in Goodman et al.,^15^ Monchalin et al.^30^ and Oelke^25^ divulged having omitted sharing their Indigenous background and certain aspects of their medical history to PHC providers, as they believed that this information would not be beneficial for their care, and worse, might only lead to discriminatory acts. Others chose to dress or behave differently in front of PHC providers to gain respect.^32^ Indeed, one First Nations participant living in a remote community elaborated in Browne and Fiske: 

It seemed like any time I go to a doctor I would have to be well dressed. I have to be on my best behaviour and talking and I have to sound educated to get any kind of respect…. If I was sicker than a dog and if I didn’t want to talk and I didn’t care how I sounded or whatever, I’d get treated … like lower than low. But if I was dressed appropriately and spoke really well, like I usually do, then I’d get treated differently…. But why do I have to try harder to get any kind of respect? You know, why do I have to explain?^32^^,p.135 ^

In certain cases, Indigenous patients delayed seeking care as long as possible to prevent being subjected to traumatic and discriminatory experiences.^15^,^25^ They sought health care only when their illness or symptoms had become serious, and they were left with no choice.^15^,^25^ Many participants from both urban and rural or remote regions admitted to distrusting PHC providers.^13^,^24^,^26^,^34^ However, Inuit and First Nations patients residing in rural or remote communities expressed significant concerns about whether providers were adequately protecting their privacy and confidentiality.^14^,^22^,^24^

When comparing the PHC experiences of First Nations, Mtis and Inuit participants in urban and rural or remote settings, we found very limited differences. As demonstrated above, similar to Indigenous patients living in urban areas, rural or remote participants also faced discriminatory attitudes and dismissive and judgmental care, forcing them to develop strategies for coping with such maltreatment. One particular geographical difference, however, was the fear of privacy and confidentiality breach. Although one participant in the study by Bucharski et al.,^37^ which included First Nations and Mtis women in an urban setting, expressed their concern about privacy and confidentiality, multiple First Nations, Mtis and Inuit participants in rural or remote locations highlighted this fear. This concern may be more significant for residents of close-knit, small communities, as are often found in rural or remote locations. For these participants, PHC providers who were not considered “locals” were at times preferred, since they did not know anyone from the community and/or they would only be temporarily working in the community.^14^

***Synthesized finding three: structural and practice issues***

Synthesized finding three highlights issues related to the PHC system’s structure and practices that led Indigenous people to experience inaccessible and incomplete care. Four categories and 32 findings formed the basis of this metasynthesis. 

Our review found that major shortages of PHC providers existed across Canada. As a result, the Indigenous patients in the studies we reviewed who lived in both urban and rural or remote settings experienced lack of continuity of care, inaccessibility, short visits and inadequate health teaching and promotion. Many First Nations and Mtis people who lived in cities did not have a family doctor; hence, they most often opted to visit walk-in clinics where various physicians rotate to cover the hours, and patients did not necessarily see the same physician during all their visits.^25^,^35^ Establishing a therapeutic physician–patient relationship may be impossible in such brief encounters. This issue was even more problematic in rural and remote communities, where the transiency of PHC providers is prominent, and their recruitment and retention are challenging.^22^,^27^,^32^,^35^ Some First Nations and Mtis participants in Jacklin et al. “felt that once doctors gain experience, ‘they want more money here, and if they don’t get it, they quit and move on.’”^35^^,pp.109-110^

Additionally, Inuit and First Nations patients who lived in rural or remote regions could not easily access certain medical care and preventive services.^12^,^14^,^34^,^39^ Minimal or no time was dedicated to health teaching or promotion, especially in a manner that was culturally appropriate.^14^ Indeed, as one of the Inuit participants in Fraser and Nadeau confirmed, 

If I was diabetic, for example, I would need information, what can I eat and what can I not. My Grandmother, they did not give her any ideas what she can eat and what she cannot do…. They need to have examples, recipes, and take less salt and sugar. And, how to make bannock. Like when you make spaghetti, use the whole wheat spaghetti. All those nutrition information. People need encouragement.^14^^,p.292^

Though health promotion materials, such as brochures and videos, may be available, First Nations and Mtis participants in Oekle^25^ further highlighted the absence of culturally adapted verbal and visual teachings. One participant reported, “The prevention services that are available for First Nations are what’s ever in the hype for the White crowd. So if it’s a White problem, a White prevention problem, those are what’s available.”^25^^,p.147^ Also, visits of First Nations and Mtis patients with PHC providers in metropolitan, rural and remote areas were commonly described as “rushed,” there being “never enough time,” “a race to fit as much patients as possible” and “similar to an assembly line.”^23^,^31^,^33^,^35^ For this reason, many felt that their needs and concerns were not entirely addressed.^33^,^35^

In regard to other geographical considerations, despite the differences of PHC services offered in urban and rural or remote settings, PHC structure and practices in all three settings similarly affected the accessibility of care experienced by Indigenous people. For instance, in rural or remote locations, hospitals and specialized care did not necessarily exist. PHC providers within these settings therefore generally assumed an expanded role to offer additional services to community; however, this had its limits, as certain diagnostic tools and specialists were only available in the major cities.^12^,^14^ In urban areas, First Nations and Mtis people encountered comparable accessibility challenges, including the lack of PHC services for children and youth, and mental health support.^25^

***Synthesized finding four: recommendations***

Synthesized finding four focussed on Indigenous patients’ recommendations for greater emphasis on culturally sensitive empathic care, recruitment of Indigenous PHC providers, accessibility and health teaching and promotion. This last metasynthesis was created from five categories and 25 findings. 

Numerous First Nations, Mtis and Inuit participants emphasized the importance of cultural sensitivity and empathy, indicating that it is paramount that all PHC providers and staff are familiar with Indigenous history and practices.^24^,^33^,^37^ They expressed the idea that only through education would providers and staff start to be empathic and respectful towards Indigenous peoples.^13^,^37^ Besides provider–patient interactions, cultural sensitivity could also be conveyed in the design of the physical spaces where PHC services are delivered. Participants suggested that incorporating Indigenous symbols or art onto the walls of the clinic could provide a more welcoming environment for patients.^29^

Participants also suggested that greater funding should be allocated to recruiting PHC providers and staff, particularly those with an Indigenous background.^12^,^23^,^25^,^34^-^36^ As one participant explained, “I just think they need to have more Native doctors and nurses ... for Aboriginal peoples to feel comfortable ... or people that are experienced in Aboriginal culture. It would be nice to have our own Aboriginal people running it.”^23^^,p.83^

Furthermore, there is a great need to enhance health teaching and promotion in all PHC settings.^14^,^25^,^33^,^35^ Health teaching and promotion must also appropriately consider the cultural context and challenges of Indigenous peoples for these to be perceived as beneficial.^25^

Lastly, when geographical differences between urban and rural or remote settings were examined, minor nuances were noticed. Although recommendations for culturally sensitive empathic care, recruitment of Indigenous PHC providers, improved accessibility and health education were common across First Nations, Mtis and Inuit participants from urban and rural or remote regions, certain recommendations were given more emphasis within one particular setting. For example, the need for culturally sensitive empathic care and recruitment of Indigenous PHC providers was pointed out more by First Nations and Mtis participants residing in the urban areas than those in rural or remote communities, who mostly emphasized suggestions for accessibility and health teaching and promotion.

## Discussion

The purpose of this qualitative systematic review was to explore Indigenous people’s experiences in Canada with PHC services, determine urban versus rural or remote differences and identify recommendations for quality improvement. Three major synthesized findings were revealed—supportive and respectful experiences, discriminatory attitudes and systemic challenges faced by Indigenous patients—along with one synthesized finding on their specific recommendations. 

The conflicting PHC experiences of First Nations, Mtis and Inuit participants, wherein instances of supportive and respectful interactions were revealed while discriminatory attitudes and systemic barriers simultaneously exist, attest to the multifaceted complexity of the situation. The interplay between systemic, institutional and interpersonal factors may have influenced these conflicting PHC experiences. The historical and intergenerational traumas of colonization, forced assimilation and residential schools continue to leave a lasting effect on the health care system, contributing to systemic discrimination that is ingrained within Canada’s health care policies and structures. The policies and structures of the health care system often reflect historical biases and stereotypes rooted in the colonial era and the legacy of residential schools. These biases manifest in policies that fail to adequately address the unique health challenges faced by Indigenous populations, resulting in unequal access to health care resources and services. 

Additionally, there is limited Indigenous representation in health care policy making and leadership. This absence of perspective leads to a health care system that often does not fully understand or prioritize the health needs of Indigenous communities, further alienating them from the system. Although at the institutional level some organizations have invested in cultural sensitivity and antiracism training for health care providers, which can result in more positive experiences for Indigenous patients, individual health care providers within these organizations may still hold conscious or unconscious biases against Indigenous peoples, which can negatively affect the quality of care received. 

In sum, the disparity in PHC experiences among Indigenous communities arises from a multifaceted set of conditions that operate at various levels. While systemic issues such as discrimination and racism can lead to negative experiences, targeted interventions and personal relationships can sometimes result in positive interactions. Therefore, efforts to improve PHC health care for Indigenous people in Canada need to be comprehensive, multipronged and culturally sensitive to effectively address this complex situation.

Indigenous people in this review valued safe, accessible and respectful care, aligning with their basic human rights as outlined in the United Nations Declaration on the Rights of Indigenous Peoples^41^ and the Truth and Reconciliation Commission (TRC) of Canada’s calls to action.^42^ Canadian governments and other sectors are nowhere near fulfilling these calls to action,^43^ particularly in the domain of health. At the current pace, completing all the calls to action will take until 2065.^43^ This shortcoming is particularly evident in our review; significant findings from most of the included articles illustrated considerable discrimination, racism and maltreatment of Indigenous peoples. Synthesized findings two and three echoed these unjust experiences that Indigenous patients had to face (and potentially continue to face).

The discrimination and racism faced by the Indigenous people in this review negatively affected their overall health and well-being. While accessing PHC, they often felt uncomfortable and judged due to providers’ negative stereotypes of Indigenous people. These attitudes, along with dismissive care and maltreatment, caused Indigenous people in the studies reviewed to avoid seeking care, exacerbating medical symptoms and potentially leading to severe complications or death. 

Similar findings in other studies show that past experiences of discrimination and racism made Indigenous people more likely to avoid medical assistance, contributing to unfavourable health outcomes.^3^^,44^ The life expectancy of Indigenous people is five years less than that of the general population.^3^ Additionally, the prevalence of infectious diseases, chronic conditions and mental health disorders as well as infant mortality rates among Indigenous populations in Canada are significantly higher compared to non-Indigenous Canadians.^3^ These disparities were further exacerbated during the pandemic, particularly for Indigenous people in rural and remote communities, who contracted COVID-19 at rates three to four times the national average—rising to seven and eight times in some weeks.^45^

In this review, First Nations, Mtis and Inuit participants living in rural or remote locations were also more likely to experience maltreatment and dismissive care as well as issues with privacy, confidentiality and accessibility.^12^-^15^,^22^,^24^,^34^ These particular issues could be attributed to the close-knit nature of small communities and the structural barriers associated with the lack of health care infrastructure within these areas. Even though we identified 10 studies of rural and remote regions, there were still limited findings on Indigenous people’s PHC experiences in such regions, which prevented a deeper analysis of geographical considerations. The inclusion of participants from diverse geographical settings, however, adds another layer of complexity and richness to the findings, as it allows for a more nuanced understanding of how location may impact health care experiences. Hence, more research on PHC experiences of Indigenous peoples living in rural or remote communities is required to comprehensively understand the challenges they encounter. 

Overall, the synthesized findings of this review emphasize the urgent need to address longstanding discrimination and racism, while also advocating for the implementation of sustainable changes to prevent further endangerment of Indigenous lives in Canada. 

***Recommendations***

Indigenous patients have highlighted numerous problems with PHC services, leading to calls for changes in health care practice, structures and policy development. This includes emphasizing Indigenous culture in training, improving cross-cultural communication and prioritizing education to reduce negative experiences, all of which are in line with the TRC calls to action numbers 23 and 24.^42^,^46^ Despite an increase in cultural competency and antiracism training,^47^ there is still a need to increase the methodological rigour and standardization of such training, as well as to examine their long-term effects while stressing Indigenous community partnerships.^46^,^48^ Health care providers should also practise some form of self-reflection, such as journalling or meditation, to examine personal biases.^49^ This approach, aligned with cultural humility principles, teaches providers to defer to clients as experts in their own culture, creating a safer, nonjudgmental environment with the voices of Indigenous patients at its forefront.^49^

However, the focus of change should not be solely on health care practice and providers. Systemic transformation, including more funding and support for Indigenous communities, must happen concurrently in order to establish meaningful traction towards better patient care. There is a nationwide shortage of Indigenous PHC providers and staff that requires immediate attention. As emphasized in the TRC calls to action, “We call upon all levels of government to increase the number of Aboriginal professionals working in the health-care field [and to] ensure the retention of Aboriginal health care providers in Aboriginal communities…”^42^^,p.164^ These key actors are critical in all sectors of society, from frontline and academia to research and policy development.^49^ At this point, the inclusion of Indigenous people across all sectors should be the norm, and not merely an afterthought. 

***Strengths and limitations***

This is the first qualitative review exploring Indigenous people’s experiences with PHC services across Canada, serving as a valuable guide for policy makers and health care providers to identify target areas for improvement. Only by incorporating the voices of service users into health policies and interventions will the PHC and health care system as a whole deliver services that truly and meaningfully meet patients’ and communities’ needs. However, a limitation of qualitative review stems from the pooling of findings that are context-dependent, thus potentially reducing the emphasis on important contextual factors. Nevertheless, through our use of the chosen methodology (i.e. meta-aggregation), the traditions of qualitative research were maintained, preserving the context of each study and aggregating findings into a combined whole.16 This strengthens the review’s findings, making them more appropriate for guiding policy makers and health care providers.

## Conclusion

Despite some supportive and respectful encounters with PHC providers, the majority of the experiences of Indigenous peoples were inadequate, unjust and filled with discriminatory attitudes and behaviours. Certainly, more work needs to be done before Canada meets all five core principles of the *Canada Health Act*.^7^ These principles are the basis of our health care system and should be applicable to all Canadians, irrespective of their age, gender, race and cultural background.^7^ Therefore, it is the duty of Canadian governments, other sectors and citizens to ensure that Indigenous people receive the health care they deserve.

## Acknowledgements

We acknowledge Ms. Anita Kiafar for her ideas and feedback during the initial conceptualization phase of this systematic review. While not involved in the writing, her input during discussions was appreciated.

## Funding

This research did not receive any funding.

## Conflicts of interest

The authors declare there are no conflicts of interest.

## Authors’ contributions and statement

GB, SA—conceptualization.

GB, SA—formal analysis.

GB—project administration.

GB, SA—visualization.

GB—writing—original draft.

GB, SA—review & editing.

The content and views expressed in this article are those of the authors and do not necessarily reflect those of the Government of Canada.
